# Cost-Effectiveness Analysis of a Mobile Ear Screening and Surveillance Service versus an Outreach Screening, Surveillance and Surgical Service for Indigenous Children in Australia

**DOI:** 10.1371/journal.pone.0138369

**Published:** 2015-09-25

**Authors:** Kim-Huong Nguyen, Anthony C. Smith, Nigel R. Armfield, Mark Bensink, Paul A. Scuffham

**Affiliations:** 1 Centre for Applied Health Economics, Menzies Health Institute, Griffith University, Meadowbrook, Queensland, Australia; 2 Centre for Online Health, The University of Queensland, Brisbane, Queensland, Australia; 3 Queensland Children’s Medical Research Institute, Brisbane, Queensland, Australia; Institute for Health & the Environment, UNITED STATES

## Abstract

Indigenous Australians experience a high rate of ear disease and hearing loss, yet they have a lower rate of service access and utilisation compared to their non-Indigenous counterparts. Screening, surveillance and timely access to specialist ear, nose and throat (ENT) services are key components in detecting and preventing the recurrence of ear diseases. To address the low access and utilisation rate by Indigenous Australians, a collaborative, community-based mobile telemedicine-enabled screening and surveillance (MTESS) service was trialled in Cherbourg, the third largest Indigenous community in Queensland, Australia. This paper aims to evaluate the cost-effectiveness of the MTESS service using a lifetime Markov model that compares two options: (i) the Deadly Ears Program alone (current practice involving an outreach ENT surgical service and screening program), and (ii) the Deadly Ears Program supplemented with the MTESS service. Data were obtained from the Deadly Ears Program, a feasibility study of the MTESS service and the literature. Incremental cost-utility ratios were calculated from a societal perspective with both costs (in 2013–14 Australian dollars) and quality-adjusted life years (QALYs) discounted at 5% annually. The model showed that compared with the Deadly Ears Program, the probability of an acceptable cost-utility ratio at a willingness-to-pay threshold of $50,000/QALY was 98% for the MTESS service. This cost effectiveness arises from preventing hearing loss in the Indigenous population and the subsequent reduction in associated costs. Deterministic and probability sensitivity analyses indicated that the model was robust to parameter changes. We concluded that the MTESS service is a cost-effective strategy. It presents an opportunity to resolve major issues confronting Australia’s health system such as the inequitable provision and access to quality healthcare for rural and remotes communities, and for Indigenous Australians. Additionally, it may encourage effective health service delivery at a time when the healthcare funding and workforce capacity are limited.

## Introduction

Indigenous Australians experience some of the highest levels of ear disease and hearing loss in the world, with rates of up to 10 times more than those for non-Indigenous Australians. The prevalence of ear disease in some Aboriginal and Torres Strait Islander communities has been reported to be as high as 91% with unilateral otitis media with effusion (OME) in 31%, bilateral OME in 26% and chronic suppurative otitis media (CSOM) in 15% of children [1]. The World Health Organization defines a prevalence of CSOM in a given population of greater than 4% as a massive public health problem [2].

Ear diseases, including recurrent acute otitis media (AOM), OME and progression to CSOM, cause high rates of conductive hearing loss [3]. A cascade of follow-on effects occurs including detrimental effects on social and emotional wellbeing [4,5], behaviour [5], educational outcomes [6], and employment [7,8]. Hearing loss has also been implicated as a factor in criminal misadventure [9].

Screening, surveillance and timely access to specialist ear, nose and throat (ENT) services are key components in detecting and preventing the recurrence of AOM and OEM, the progression to CSOM and in ultimately reducing hearing loss [10]. This is particularly important for Indigenous children because compared with their non-Indigenous counterparts, they are at an increased risk of experiencing ear disease at an earlier age and with recurrence [11] leading to increased risk of progression to serious ear disease and conductive hearing loss. They are also less likely to present with a complaint of hearing loss or discharging ears [7,8].

To this end, a collaborative, community-based mobile telemedicine-enabled screening and surveillance service (MTESS) is being trialled. Operating from the Cherbourg Community Health Service, it serves Queensland’s third largest Indigenous community and children in the greater South Burnett area. The MTESS service provides screening and surveillance at schools and aims to facilitate the early identification and monitoring of Indigenous children at risk of developing ear disease. While screening and surveillance services are essential to identify children with ear disease, used alone they do not lead to a reduction in prevalence [12]. Rather, screening and surveillance must be integrated with appropriate treatment services. Thus, rather than being a stand-alone service, the MTESS service integrates with an existing community-based ear health program, the Deadly Ears Program, which provides outreach surgical services and referral to a general practitioner (GP) and tertiary level treatment services when indicated [13,14].

The primary goal of the MTESS service is to increase the screening and surveillance rate of children to greater than 90% within the given community and subsequently to improve treatment rates and hearing outcomes. This is based on the notion that taking a fully equipped mobile telemedicine-enabled screening and surveillance service to children whilst they attend school or day care will result in greater screening and surveillance rates than the alternative, in which either the Indigenous health worker (IHW) must set up makeshift clinics in schools, or parents must travel to the local hospital for screening.

As the MTESS involves additional investment in capital and human resources, it is essential to ensure scarce resources are allocated efficiently. This study aimed to assess the cost-effectiveness of a supplemental mobile telemedicine-enabled ear health screening and surveillance service for Indigenous children living in regional communities compared with the existing outreach screening and surgical service alone.

## Method

Cost-utility analysis was the chosen analytical method because the outcome measure, quality-adjusted life year (QALY), quantifies the burden of disease on patients by adjusting life length with a functionality index [15]. Although ear diseases and deafness rarely shorten life expectancy, hearing loss affects the patient’s ability to fully function for the rest of their life. Additionally, a QALY does not require age weighting (i.e. old and young patients are treated equally), and it is relatively easy to incorporate in the mathematical model for the analysis.

A life-time Markov cohort model with a 12-month-cycle was used to compare the two different screening strategies against ear disease and hearing loss. The model, programmed in TreeAge 14 software (Data^TM^, TreeAge Software Inc.), used a matrix of transition probabilities between different health states to simulate the effect of screening and surveillance strategies on the progression of ear disease and hearing loss. The long time horizon, covering patients aged three years and extending into their adulthood, reflected the at-risk time period (i.e. 3–18 years) and the sustained long-term commitment required to affect ear disease related hearing outcomes of Indigenous children. It also incorporates the effects of early hearing loss on education, which in turn affects employment opportunity in adulthood, and social and emotional wellbeing within and beyond childhood. The 12-month cycle aligned with the average screening schedule for Indigenous children in the Deadly Ears Program and the MTESS service initiatives.

### Screening and surveillance strategies

Two screening strategies were compared in the model: the Deadly Ears Program, and the supplemental mobile telemedicine service (MTESS). The Deadly Ears Program was the existing outreach service in the South Burnett community prior to the introduction of the mobile telemedicine service (MTESS).

### Deadly Ears Program

The Deadly Ears Program surgical outreach service to South Burnett began routine operation in 2008 and focuses on local treatment by ENT specialists visiting bi-annually from a tertiary paediatric hospital in Brisbane. Routine ear screening and surveillance is provided by a dedicated, full-time senior IHW specifically designated to ear health. The IHW provides voluntary screening assessment at the local community hospital. *Ad hoc* screening assessments are conducted in schools when the IHW is available. Those children who fail assessment are referred to the surgical outreach clinic, which is a ward in a local hospital temporarily converted into a surgical space. The clinic includes a medical review of each child that is referred with a follow-on referral to local GPs for medical treatment (primarily antibiotics) where indicated. When surgical intervention is indicated, children are booked in for subsequent surgery, either at outreach, at the nearest general hospital or at the tertiary children’s hospital, as appropriate. For the surgical component, all the required consumables and surgical equipment (e.g. anaesthetic machine, patient monitor, etc.) are brought along by the surgical visiting team, who come from tertiary services in Brisbane for each four-day outreach clinic.

### Mobile telemedicine-enabled screening and surveillance service (MTESS)

The supplemental mobile telemedicine service (MTESS), integrated with the outreach surgery clinic, is delivered by an IHW with advanced hearing-health training. The services are performed in a mobile van custom fitted with a video-otoscope used to capture high quality still images of the tympanic membrane, a typanometer to test middle ear function, and an audiometer to assess hearing. The van is driven to local community day care centres, primary schools and high schools to conduct health screening assessments of Indigenous children. Additional visits can be offered for young children identified as not attending day care or school. Following assessment, results are uploaded to a secure database and reviewed by the specialist ENT team in Brisbane which provide a diagnosis and treatment plan (i.e. online assessment). Surgical components of this treatment plan are then referred back to the Deadly Ears Program surgical outreach clinic. Medical components of the treatment plan are referred to local community GPs (details of the MTESS service are reported in detail elsewhere) [13,14]. Of note, the average screening rate achieved in the community after the introduction of the MTESS service reached 75–90%.[13]

The primary differences between these two strategies were the screening and surveillance rate, the subsequent treatment rate achieved, and the screening cost associated with each option. All other aspects of ear disease and hearing loss treatment, related hearing outcomes and costs, were identical.

### Model structure

**Fig 1:**
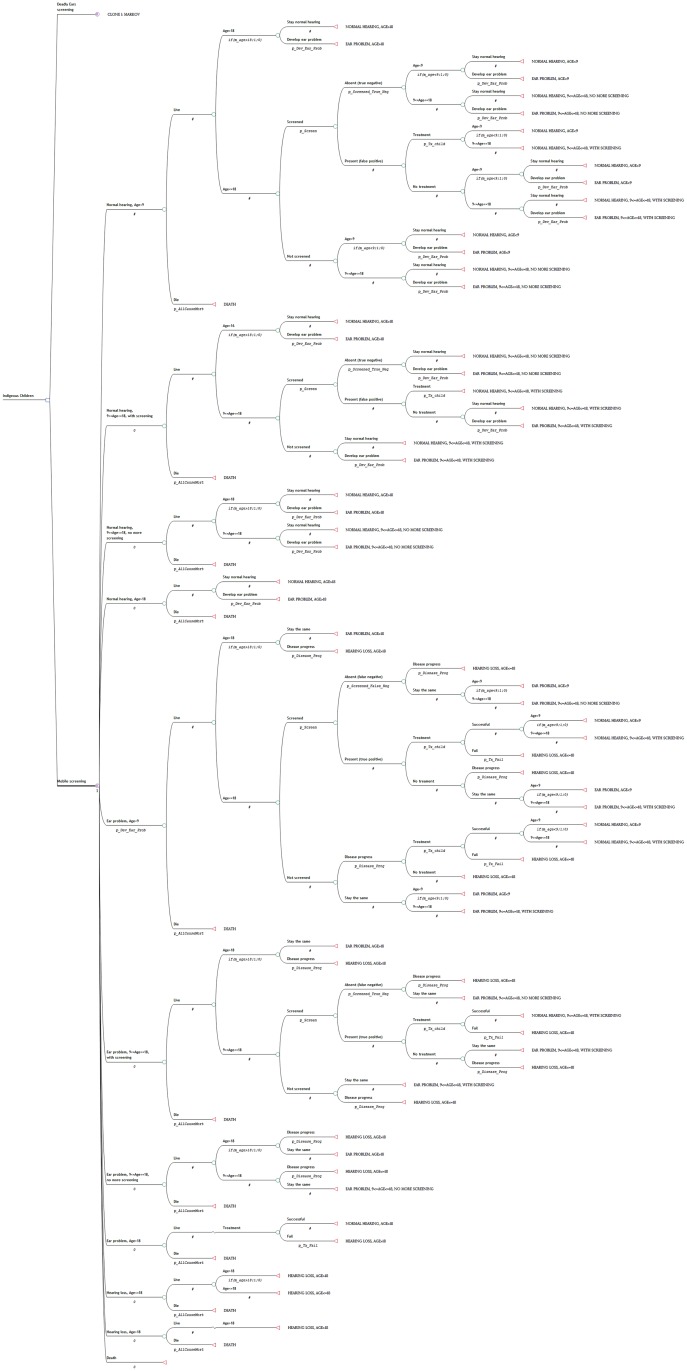
Markov states used for each of the options analysed in the decision analytic model.

For each strategy (Deadly Ears Program and MTESS), a four health state Markov cohort model using 12-month cycles was developed to calculate the outcomes of screening and treatment and the cost (Fig 1). The starting age for screening was three years old. The model terminated when the cohort reached the average age of 50, which is the age when most people would develop a hearing problem.

All patients in the cohort entered the model from one of the two health states, *healthy* (no hearing or ear problem) and *ear problem*, from which they received screening. Patients in these two health stats received screening because ear disease can be latent and may go undetected unless the child is screened or has a GP check-up. If ear diseases or hearing problems were detected, the child could receive treatment and regain the healthy status. However, not all children received treatment [7], and some treatments were unsuccessful [16,17], which led to *hearing loss* or a continued ear problem. Some children with deafness received hearing loss support, either in the form of normal hearing aid equipment or a cochlear implant, and they remained in this health state permanently. When a child reached the age of nine years, an age whereby it is generally accepted that Eustachian tube development is complete and risk of middle ear disease is reduced, and the screening or surveillance result was negative (i.e. no ear disease detected), the patient was discharged from the program. When children reached the age of 18 years, they no longer received screening but continued to move between the four specified health states. Mortality could occur at any point time during the model duration but was not altered by the progression of ear disease.

### Data and assumptions

#### Probabilities

The probability of participating in a screening program varied according to the strategy under analysis (either Deadly Ears Program or MTESS). After this point, the model for both strategies was identical incorporating the probability of ear disease in the population, the probability of patients accessing and obtaining treatments, the probabilities of false detection, the probability of treatment failure, and the probabilities of hearing loss associated with and without treatment.

According to the Queensland primary health care patient information and recall system (FERRET) for rural and remote Indigenous communities, screening rates of the Deadly Ears Program were estimated to be 35% in 2008 (for an estimated 1,000 population). This rate was used to approximate the probability of being screened under Strategy A (Deadly Ears Program). Elliot and colleagues reported 745 parent consent to MTESS service screening via 16 of the 19 South Burnett area schools (for a population of 980) [14]. This translated to a coverage (screening) rate of 76%. Since then, statistics from the screening and surveillance service suggest that the coverage has increased to approximately 80% of the school-age population. This rate informed the probability of being screening under the MTESS service.

The probability of developing ear disease was age-dependent, which reflected differences in prevalence (and incidence) rates in acquiring ear and hearing problems amongst children aged 3–18 and adults. The prevalence data was extracted from the Australian Aboriginal and Torres Strait Islander Health Survey 2012–13 [18]. We did not differentiate between males and females in the model.

For the probability of receiving treatment after being diagnosed, data from a recent review of ear health and hearing for Indigenous Australians was used. A study reported 16% of Indigenous children living in the remote areas with ear or hearing problems did not receive treatment compared with 9% of those with ear or hearing problems living in non-remote areas [7]. In the absent of further information, the weighted average of these two values was used as the probability of not getting treatment, with the weights were population shares of remote (75%) and non-remote areas (25%).

To reflect the current medical practice, the model assumed two treatment options: medical and surgical. Medical treatment is indicative of the recommended use of antibiotics for recurrent AOM, OME and CSOM by national guidelines, primarily high-dose, long-term antibiotics. Surgical review and possible treatment is usually recommended for patients with (i) OME and bilateral hearing loss for 3-months with failure of an appropriate antibiotic regimen or if severe retraction of the ear drum is present, (ii) frequent painful AOM, and (iii) significant conductive hearing loss [19]. The probability of patients receiving either of these interventions in each cycle was reflective of the level of medical and surgical activity possible for the given population with the resources stipulated in this analysis. The Deadly Ears Program provided a total of 200 medical reviews and 40 surgical procedures. This represented a five to one ratio or a probability of surgical intervention of 0.2.

For the probability of hearing loss associated with either medical or surgical treatment, there is no up-to-date study identified with information relevant for the Australian Indigenous population. Similarly, there is limited literature available to inform the probability of hearing loss for Indigenous children in the absence of treatment, the rates of screening failures (either false negatives or positives), and the percentage of Indigenous patients receiving hearing aids for deafness. Therefore, these parameters were sourced from best knowledge of experts in the field and relevant officers involved in the screening programmes. When parameters are equivocal as such, conservative estimates are selected and sensitivity analyses over a wide range of estimates are performed.

Specific Indigenous childhood and adult mortality rates for Queensland [20] were used to account for the proportion of patients that were deceased at the end of each cycle. Of note, no additional mortality was assigned to treatment, or lack of treatment, for ear diseases.

All transition probabilities are reported in Table 1.

**Table 1:** Transition probabilities.

	Base case	Sensitivity	Sources
Developing ear problems	Age dependent	Beta distribution	ABS 2014 data
Being screened by Deadly Ears	0.39	Beta distribution	Queensland Ferret database
Being screened with MTESS service	0.80	Beta distribution	Elliot et al 2009;
Screening returns false negative (diagnosed no ear problem given having ear problem)	0.05	Triangular distribution, range 0.02–0.20	Expert opinion
Screening returns true negative (diagnosed no ear problem given normal hearing)	0.90	Triangular distribution, range 0.80–0.95	Expert opinion
Getting treatment if diagnosed or have obvious sign of ear problem	0.86	Beta distribution	Burns et al 2013; ABS 2014
Receive medical treatment (instead of surgical treatment)	0.80	Triangular distribution, range 0.70–0.90	Assumption
Treatment failure (both medical and surgical)	0.10	Beta distribution	Expert opinion
Progression from ear problems to hearing loss without treatment	0.10	Beta distribution	Expert opinion
Getting hearing aids in Indigenous children	0.05	Triangular distribution, range 0.02–0.15	Expert opinion
Getting hearing aids in Indigenous adults	0.35	Triangular distribution, range 0.10–0.50	Expert opinion

Abbreviation: MTESS = mobile telemedicine-enabled screening and surveillance;

#### Cost data

All costs included in the model are described in 2013–14 Australian dollars. Vehicle, equipment, installation, consumables and staff costs are valued at market prices (1st January 2015, AUD 1 ≈ USD 0.81, €0.69, £0.53) [21]. All vehicle and equipment capital costs are converted to annual equivalent costs at an annual rate of 5% with depreciation and zero salvage value for the expected life of the item. The total annual cost for the Deadly Ears Program screening service includes costs for screening equipment, one IHW, an ‘average’ bundle of consumables and travel costs. The travel costs were equivalent to the travel distance required to cover 35% of the given population (2,533 enrolled children) with an average weekly millage of 75km. For the coverage of 887 children (35% of 2,533), the average cost per screen was then calculated as the total cost divided by the number of screens, or A$88 per screening (Table 2).

**Table 2:** Costs of screening, both strategies.

	Unit	Unit cost	AEC
**Deadly Ears Program**			
**Fixed costs**			
Screening equipment	5 years	$5,852	$1,352
Carry cases	2 years	$504	$271
**Variable costs**			
Health worker	1 FTE	$73,238	$73,238
Consumables	1 year	$1,076	$1,076
Mileage reimbursement	3,075km^a^	$0.8	$2,306
**Total cost**	887 children^b^	**$88**	**$78,243**
**MTESS Service**			
**Fixed costs**			
Van, fit-out and equipment	5 years	$192,298	$44,416
Garage	5 years	$23,256	$5,372
Database costs	5 years	$50,236	$11,603
**Variable staff costs**			
Health worker	2 FTEs	$73,238	$146,746
Senior ENT surgeon	169 hours^c^	$121	$20,495
**Variable travel, network and consumable costs**			
Petrol	6,150 km^d^	$0.8	$4,613
Broadband wireless Internet access	12 months	$165	$1,980
Clinical supplies	1 year	$1,246	$1,246
**Total cost**	2026 children^e^	**$117**	**$236,200**

Abbreviation: ACE = annual equivalent cost; ENT = ear, nose, throat; FTE = full time equivalent;

^a^Estimated travel distance required to cover 44% of the given population with an average weekly millage of 75km;

^b^Assuming 35% of the estimated 2,533 registered children in the community are screened every 12-months;

^c^Estimated total time to review 2026 screening assessments conducted in 1-year with an average review time per screen of five minutes;

^d^Estimated travel distance required to cover 80% of the given population with an average weekly millage of 75km;

^e^Assuming 80% of the estimated 2,533 registered children in the community are screened every 12-months

The annual cost to provide a MTESS service was also calculated along with the associated average cost per screen. It is assumed that the MTESS service covered the same population (2,533 children) with 80% screened annually. The calculated annual equivalent cost (AEC) for all equipment was combined with staff, consumable, and infrastructure costs based on the costs incurred during a six-month feasibility trial [14]. Fixed costs included the cost of the van, fit-out and all required equipment plus purchase (or construction) of a garage/shed to house the van. Maintenance costs for the van included annual registration, insurance, running and mechanical maintenance costs. Staff costs included a part-time service manager, ENT surgeon time for online assessments and a full-time IHW that runs the van and performs screening and surveillance assessments. Database development, maintenance and renewal were included as an additional AEC, which reflected the initial and ongoing maintenance investment required for the database. The cost of wireless broadband internet connectivity for the van was also included (Table 2).

Treatment costs were applied separately for children (up to 18 years) and adults. Medical treatment includes GP visits for diagnosis and average appropriate dose of antibiotics of up to A$82 per child, or A$91 per adult. Surgical services were calculated separately for these two age groups. When a child enters adulthood, it is assumed that the outreach surgical service is no longer appropriate. The patient instead receives treatment from hospital after having medical reviews (from a GP and a specialist, for treatment plan). The hospital surgical cost was approximated using DRG-D06Z (middle ear procedure), or A$5,757 per case (AR-DRG v6) [22]. This adds up to an estimate of A$6,021 per surgical treatment.

The cost break-down for the outreach surgical service (applied for children up to 18 years) is summarised in Table 3. It was assumed an appropriate ward area in a local community hospital was available to conduct a temporary surgical clinic at no cost. Information on permanent staff requirements for the service was included along with the cost of specialist surgical staff required to complete 160 operations in a calendar year. An ‘average’ bundle of surgical and anaesthetic consumables typically required was also included. This results in a cost of A$2,369 per surgery.

**Table 3:** Costs for surgical treatment in outpatient clinic.

	Unit	Unit cost	AEC
**Fixed costs**			
Anaesthetic machine	5 years	$68,000	$15,706
Anaesthetic monitor	5 years	$38,000	$8,777
Additional anaesthetic equipment	2 years	$2,611	$1,404
Patient monitor	3 years	$9,685	$3,556
Miscellaneous equipment	3 years	$9,011	$3,309
Surgical instruments	10 years	$74,332	$9,626
Microscope	10 years	$14,497	$1,877
Sterilizer	3 years	$6,540	$2,402
Carry cases	2 years	$1,847	$993
Clinic instruments	5 years	$2,086	$482
**Variable staff costs (Queensland Health certified enterprise bargaining agreement 2012)**			
Nurse manager (per annum)	1 FTE	$98,153	$98,153
Clinical nurse	1 FTE	$79,992	$79,992
Senior ENT surgeon	128 hours	$121	$15,523
ENT registrar	128 hours	$83	$10,574
Senior anaesthetic consultant	128 hours	$118	$15,066
Anaesthetic registrar	128 hours	$80	$10,236
Anaesthetic technician	128 hours	$56	$7,198
Scrub/scout nurses	128 hours	$50	$6,365
Recovery room nurse	128 hours	$50	$6,365
**Consumables**			
Anaesthetic consumables			$8,700
Anaesthetic drugs			$8,700
Surgical consumables			$15,500
**Variable travel and accommodation costs**			
Truck rental (4 x 5 day trips)	20 days	$189	$3,780
Passenger van rental (4 x 5 day trips)	20 days	$137	$2,740
Petrol (4 x 560km);	2,240 km	$0.8	$1,680
Accommodation (4 x 4 night stays for 12 single rooms)	192 nights	$110	$21,120
Meal allowance (4 x 5 day trips for 12 people at $70/day)	240 days	$80	$19,200
**Total annual cost**			**$379,023**
**Cost per surgery (estimated 160 cases performed per year)**			**$2,369**

Abbreviation: ACE = annual equivalent cost; ENT = ear, nose, throat; FTE = full time equivalent;

Hearing aids and cochlear implants are hearing prostheses used by people with hearing loss to aid communication [8]. While hearing aids only make sound louder, a cochlear implant bypasses the damaged sensory cells of the cochlea, replacing ‘acoustic hearing’ with ‘electric hearing’ through the implant. The annual cost for hearing aids and associated services were estimated to cost approximately A$1,606 per patient per annum [8]. The cost of a cochlear implant was sourced from the AR-DRG v.6: DRG-D01Z = A$32,714 [22]. It is assumed that the implantation would last for 15 years with negligible maintenance costs. This translates to an annual cost of A$3,152, using a 5% amortisation rate.

Other costs include special supports at school (e.g. teacher aid support, teacher education programs and an appropriate facility, etc.) and the economic cost of reduced productivity and income due to a hearing problem. It is reported that of the people with hearing problems aged 15–64 years, 55.6% reported being in paid work compared with 62.4% of people without hearing problems. This translates to a standardised difference of 11% employment opportunity and income, which was used to approximate the income loss due to hearing-related reduced productivity. This cost only applies when the patient reaches 16 years of age (Table 4).

**Table 4:** Other costs.

	Base case	Sensitivity	Sources
**Treatment costs (ear problems)**			
Medical treatment for children	$82	Gamma distribution; range ±20%	2 GP visits plus appropriate dose of antibiotics for children
Medical treatment for adult patient	$91	Gamma distribution, range ±20%	2 GP visits plus appropriate dose of antibiotics for adult
Surgical treatment for children (outreach clinic)	$2,369	Gamma distribution; range ±20%	See Table 3
Surgical treatment for adult	$6,021	Gamma distribution; range ±20%	1 GP visit plus 1 specialist visit, and cost of surgical treatment sourced from AR-DRG v.6
**Costs associated with hearing loss (monthly)**			
Non-cochlear aid for hearing loss	$1,606	Gamma distribution; range ±20%	Access Economics 2005
Cochlear implant cost	$3,152	Gamma distribution; range ±20%	Access Economics 2005, AR-DRG v.6
Education cost for children with hearing loss	$7,116	Gamma distribution; range ±20%	Access Economics 2005
Reduced income due to hearing loss in adults	$6,681	Gamma distribution; range ±20%	Access Economics 2005

Abbreviation: GP = general practitioner; AR-DRG = Australia refined diagnostic related group

#### Utilities

Quantitative information on the effects of hearing lost on quality of life were obtained from the literature (Table 5). All patients who developed hearing loss in the model were assumed to have at least moderate hearing impairment with a utility of 0.677 [23,24]. Utility weights for adults in perfect health (no hearing loss or ear problem) was sourced from a more recent quality of life study (0.95) [25], which estimated the Australia utility weight using the EuroQol EQ-5D 3 level version (EQ-5D-3L) instrument. It is assumed children in perfect health enjoy no loss of quality of life per life year (i.e. utility weight equals unity). In the absence of data, we calculated the utility weight for patients who developed an ear problem but had not year lost their hearing as an unweighted average of healthy and deafness.

**Table 5:** Utility weights.

	Base case	Sensitivity	Sources
Normal hearing in children	1.000	Triangular distribution, range 0.85–1.00	Assumption
Normal hearing in adults	0.900	Triangular distribution, range 0.85–1.00	Clemens et al 2014 [25]
Hearing loss	0.677	Triangular distribution, range 0.50–0.85	[23,24,26]
Ear problem in children^a^	0.839	-	Assumption
Ear problem in adults^b^	0.789	-	Assumption

^a^Average of hearing loss and normal hearing in children

^b^Average of hearing loss and normal hearing in adult

#### Analysis

All costs and health outcomes were discounted at 5% per annum. Half cycle corrections were used for both costs and outcomes across all transitions. Many of the cost and health outcome values and transition probabilities used in this model have considerable uncertainty. For this reason, one-way sensitivity analyses were performed on all key variables, using ranges identified in the literature. When parameters were equivocal, a wider range of estimates was chosen for the sensitivity analysis. Additionally, uncertainties were accounted for in a probabilistic sensitivity analysis using appropriate distributions with the distribution parameters estimated from the respective means and standard errors of the variables. Probabilities and utilities were assumed to follow a beta distribution and costs to follow a gamma distribution with standard errors equal to 20% of the base case values.

## Results

We validated the model by creating a business-as-usual scenario in which children were not screened (i.e. no Deadly Ears Program or MTESS service) and their ear problem (or lack thereof) was identified through GP visits. It is reported that 21.9% of the population had regular GP visits, and that 7% of the Indigenous population aged 15–24 had deafness, and by the age of 45–54, 17% had deafness [18]. From the model, it was estimated for the ‘no screening’ scenario, that 7.4% would have hearing loss at the age of 15 and 16.8% at the age of 45. Under the Deadly Ears Program and MTESS programmes, we estimated that the proportion of children aged 15 with deafness are 6.8% and 5.8%, respectively. By the age of 50, these rates are 16.1% and 15.9%, respectively.

Costs, outcomes and incremental cost-effectiveness ratio (ICER) estimates are presented in Table 6. The estimated cost for MTESS was slightly higher than for the Deadly Ears Program: A$6,262 versus A$6,235. However, the mobile screening program generated higher QALYs (15.94 vs. 15.90). This resulted in an ICER of A$656 per QALY gained. If the program enrols children aged four and above (instead of three years and above), then MTESS costs less (A$6,143 vs. A$6,176) and accrues more QALYs (15.85 vs. 15.81), making it the dominant strategy.

**Table 6:** Results for costs, effects and cost-effectiveness ratios (2013/14 AUD).

	Base case Starting age of screening = 3		Scenario Starting age of screening = 4	
	Deadly Ears	MTESS	Deadly Ears	MTESS
Total cost	$6,235	$6,262	$6,176	$6,143
Incremental cost		$27		-$33
Total QALYs	15.902	15.944	15.810	15.850
Incremental QALYS		0.042		0.039
Cost/QALY	$392	$393	$391	$388
**Incremental cost/QALY (ICER)**		**$656**		**Dominant**

Abbreviation: MTESS = mobile telemedicine-enabled screening and surveillance; QALY = Quality adjusted life year; ICER = Incremental cost effectiveness ratio

Table 7 shows the effect of parameter changes on the ICER. There was little variation in the results from changes in key parameters of the model. The most influential parameters include the probability of treatment failure, the screening and surveillance rate under the Deadly Ears Program, the rate of progression from ear diseases to deafness without treatment, and the utility of hearing loss. Changes in costs have relatively small effects on the ICER. The cost (and ICER) of the MTESS strategy would range from A$6,040 to A$6,242 (dominated to an ICER of A$1,681) if the screening cost ranged from A$93 to A$140 per child. Varying a large cost component, that is the surgical costs, does not alter the overall cost of each strategy significantly: both strategies would cost around A$6,000 to A$6,200.

**Table 7:** Summary of deterministic sensitivity analyses.

	Sensitivity range	ICER—lower value	ICER—higher value
**Transition probabilities**			
Being screened by Deadly Ears	0.3 to 0.6	Dominant	$11,365
Being screened with the MTESS service	0.7 to 0.9	Dominant	$1,561
Screening returns false negative (i.e. result = no ear problem given having ear problem)	0.02 to 0.2	Dominant	$1,125
Screening returns true negative (i.e. result = no ear problem given normal hearing)	0.8 to 0.95	Dominant	$3,284
Getting treatment if diagnosed or have obvious sign of ear problem	0.6 to 0.95	Dominant	$3,185
Receiving medical treatment (vs. surgical treatment)	0.7 to 0.9	Dominant	$2,300
Treatment failure	0.05 to 0.35	Dominant	$7,715
Progression from ear problems to hearing loss without treatment	0.05 to 0.35	Dominant	$6,605
Getting hearing aids in Indigenous children	0.02 to 0.15	$497	$887
Getting hearing aids in Indigenous adults	0.1 to 0.5	Dominant	$2,598
**Costs (estimated cost +/- 20%)**			
Deadly Ears screening cost	70 to 106	Dominant	$1,583
MTESS screening cost	93 to 140	Dominant	$3,341
Medical treatment for children	66 to 98	Dominant	$744
Medical treatment for adults	73 to 109	Dominant	$656
Surgical treatment for children under outreach clinic setting	1,895 to 2,843	Dominant	$1,308
Surgical treatment for adult	4,817 to 7,225	Dominant	$660
Non-cochlear aids for hearing loss	1,286 to 1,930	Dominant	$852
Cochlear implant cost	2,525 to 3,787	Dominant	$671
Education cost for Hearing Loss children	5,693 to 8,539	Dominant	$1,356
Reduced income due to hearing loss	5,345 to 8,018	Dominant	$2,373
**Utilities**			
Normal hearing in children	0.85 to 1.0	$582	$875
Normal hearing in adult	0.85 to 1.0	$596	$713
Hearing loss	0.5 to 0.85	$398	$1,828

Abbreviation: MTESS = mobile telemedicine-enabled screening and surveillance; ICER = Incremental cost effectiveness ratio. Note: Dominant = MTESS is the dominant strategy.

**Fig 2:**
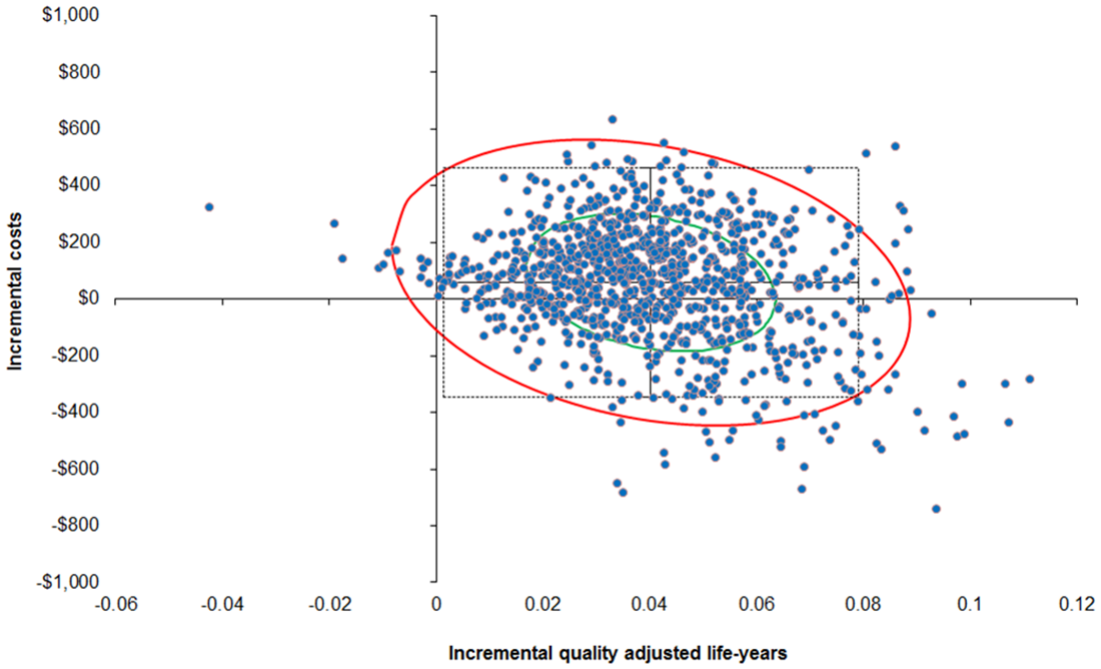
Probability sensitivity analysis results using second-order Monte Carlo simulation (10,000 draws with 95% confidence interval ellipse, with A$50,000/QALY line).

In order to more rigorously assess the effect of the uncertainty around the cost-effectiveness of the two screening strategies, a probabilistic sensitivity analysis was performed (10,000 draws with all parameters varied each draw). At the $10,000/QALY threshold, the probability that the MTESS service, when compared to the Deadly Ears Program is cost-effective is 88%. This probability increases to 98% at the threshold of A$50,000/QALY. In about one in three chances (35%), it is a superior strategy, in that the MTESS service accrues more QALYs than the Deadly Ears Program at a lower cost. The probability of the MTESS service being a dominant strategy (i.e. cheaper and more effective) is around 35% across various willingness-to-pay thresholds (Table 8 and Fig 2).

**Table 8:** Summary of distribution of probabilistic sensitivity analysis points on the cost-effectiveness plane at different willingness to pay thresholds.

		$10,000/QALY	$30,000/QALY	$50,000/QALY
I—MTESS is more costly and more effective	Less than threshold	52.81%	62.79%	63.22%
	Greater than threshold	10.60%	0.62%	0.19%
II—MTESS is more costly and less effective	Dominated	1.83%	1.83%	1.83%
III—MTESS is less costly and less effective	Less than threshold	0.02%	0.02%	0.02%
	Greater than threshold	0.00%	0.00%	0.00%
IV—MTESS is less costly and more effective	Dominant	34.7%	34.7%	34.7%

Abbreviation: MTESS = mobile telemedicine-enabled screening and surveillance; QALY = quality adjusted life years

The use of different discount rates had little impact on this overall result. At 3% discount rate, MTESS is a superior strategy, cheaper and more effective, compared to the existing Deadly Ears Program. At 7% discount rate, the ICER is A$4,109/QALY, well below the willingness-to-pay threshold of A$10,000.

## Discussion

This study is the first to examine the cost-effectiveness of supplemental mobile telemedicine-enabled screening and surveillance services for Indigenous Australian children at risk of chronic ear disease and subsequent hearing loss. Past studies in this area have focused on evaluating the quality of screening and diagnosis technologies (e.g. image quality obtained from different video-otoscopes [27], or by IHWs following specific training.^54^ or management decisions using store-and-forward telehealth [28,29]), or descriptive evaluation of changes in service volume and utilisations following different telehealth programs [13,14,30]. Our cost-effectiveness analysis provides new evidence to support the telemedicine service model for ear disease screening for Indigenous children.

This model-based analysis shows that, compared to the Deadly Ears Program, the MTESS service is cost effective, with an average 98% probability of an acceptable ICER at the $50,000/QALY threshold. The cost effectiveness arises from preventing hearing loss in the given population and subsequent reductions in associated educational support costs and hearing aids and equipment costs. This result maintains under a number of alternative scenarios including varying starting age of screening and discount rates. The most influential variables identified in the deterministic sensitivity analysis were the probability of treatment failure, the screening rate under the Deadly Ears strategy, the rate of progression to hearing loss from ear disease, and the utility weights for deafness.

The cost-effectiveness of this telemedicine model presents an important opportunity to resolve several major issues confronting the Australia’s health system including: inequality of provision and access to healthcare by rural, regional and remote Australians; access to culturally-appropriate, quality healthcare for Indigenous Australians; and effective health service delivery at a time when the health workforce capacity is limited and health funding stretched. The clinical workforce is currently centralised in metropolitan areas across Australia, primarily as a result of personal desires, proximity to general hospitals and perceived training and support requirements. Consequently, rural and remote populations lack access to the same health care options that exist for their metropolitan based equivalents. This is particularly relevant within Indigenous populations where the result of service inequities, exaggerated by cultural barriers, translates to significant disadvantage and poor health. The MTESS service may also be appropriate for other areas in Queensland, and other states and territories. Additionally, the telemedicine model may be a way of increasing coverage and reducing inequality for other services that can be delivered using the store-and-forward model, such as dermatology, endocrinology and ophthalmology.

This study was conducted from the health service provider perspective. However, the benefits of telemedicine also extend to the patient and family in the way of easier access to specialist care in the local community and the reduced the need for travel away from home. As demonstrated in a previous study, the conventional method of travelling to see a specialist is costly and stressful for the whole family [31]. The MTESS model delivers specialist services into the community with appropriate integration with local health services. Like any chronic health condition, the early identification and treatment of ear disease helps reduce hearing problems; which if left untreated, would compromise education and learning in the classroom and subsequently employment opportunities in the future. Lack of education is also attributed to issues related to delinquency.

The MTESS model was built using a collaborative process [13], bringing together community health service providers in Cherbourg and nearby towns; telemedicine experts and the ENT/hearing specialists at the tertiary hospital in Brisbane. The establishment of the service required extensive consultation with the community and the design of a service that met the requirements of all stakeholders. Since the MTESS service is a community led health service, an important success factor was the leadership and role of the senior health worker responsible for the service. For the service to be implemented in a sustainable manner, recurrent funding was secured to ensure the vehicle was maintained and a full-time staff member (coordinator) was available with appropriate backup when required. This ensured that the service could be delivered routinely throughout the region, and that continuity with key staff was maintained.

Despite these encouraging results there are a number of caveats. Of particular note, some of the evidence and information available to support this model is sub-optimal. Ear disease has been recognised as a serious problem for Indigenous people for decades; however, there remains a dearth of quality information regarding its prevalence, natural history and prognosis with different treatment options. Current work is exploring the epidemiology of ear disease and hearing loss amongst children in a remote community in the South Burnett region of Queensland, including exploring changes since the implementation of the MTESS service. Data from this work will allow more detailed analyses of outcomes and economics in the future.

Sensitivity analyses were undertaken to address the uncertainty around a range of factors; of note most factors had relatively little effect on the results, however the screening and surveillance rate and the rate of progression to deafness were the key drivers of cost, outcomes and the ICER in the model. The sustained integration of this type of screening service with community-based treatment services, and the data subsequently generated, may shed light on some of these issues.

## Conclusion

The findings of this analysis indicate that, from a health service perspective, the supplemental mobile telemedicine-enabled screening and surveillance (MTESS) service is cost effective compared to the current practice alternative alone. The benefits of telemedicine, when appropriately integrated with local health services, also extend to the patient and family in the form of preventive care. Further research is required to improve on the information used within the analytical model, to confirm or disclaim the assumptions made and to validate the internal and external consistency of the model.
