# Correction: Cost-effectiveness analysis of a mobile ear screening and surveillance service versus an outreach screening, surveillance and surgical service for indigenous children in Australia

**DOI:** 10.1371/journal.pone.0234021

**Published:** 2020-06-05

**Authors:** Kim-Huong Nguyen, Anthony C. Smith, Nigel R. Armfield, Mark Bensink, Paul A. Scuffham

After publication of this article [1], concerns were raised about whether appropriate permissions had been obtained for the use of Deadly Ears Program data in the cost-effectiveness analysis, and whether the article reported correct information about the Deadly Ears Program and its costings. In light of concerns about the Deadly Ears Program costing and resourcing details, the validity of the cost-effectiveness results was questioned. In this Correction, the authors address these concerns by providing clarifications about the approvals, study design, and data sources for this study, as well as additional sensitivity analyses that explore the impacts of some assumptions made and support the overall conclusions of the original study.

## Approvals and permissions

The authors obtained prospective ethical approval for this study from the Royal Children’s Hospital and Health Services District Ethics Committee (ethics number EC00175 and 2006/66), and from the University of Queensland Human Research Ethics Committee (ethics number 2009000278). The Office for Research at Griffith University (the corresponding author’s current institution) confirmed with the journal office that appropriate ethics approvals as required at the institutional level were in place at the time of the study. The authors also obtained letters of support in advance of the study from multiple parties, including representatives from Queensland Government and the Aboriginal and Torres Strait Islander Ear Health Program (the predecessor of the Deadly Ears Program).

Children’s Health Queensland Hospital and Health Service (CHQ HHS) have clarified with the journal office that CHQ was established as a statutory body in 2012 and that the use of Deadly Ears Program data currently requires release and authorization by CHQ; this system was not yet in place when the authors conducted the study, although ethical approval and letters of support were required at that time.

## Deadly Ears Program services

Questions were raised as to whether the frequency of Deadly Ears Program visits to Cherbourg was accurately reported. The authors clarified that staff from the Deadly Ears Program (formerly called the Aboriginal and Torres Strait Islander Ear Health Program) travelled to Cherbourg at least three times per year during the years 2006–2008 and conducted hearing tests, among other activities; this service was used as the “comparator” in the cost-effectiveness analysis, and was compared to this same service supplemented by mobile telemedicine-enabled screening and surveillance (MTESS).

By screening services, the authors referred to assessments by Deadly Ear Program staff to identify individuals who required surgery. These included an initial assessment by a clinical staff member to identify children who may have potential hearing problems, and subsequent hearing tests for such individuals conducted by an audiologist on the team.

Concerns were raised that the Deadly Ears Program services were not accurately described in the article, specifically that this program did not provide screening and surveillance service in the Cherbourg community during the study period but rather only provided Ear, Nose, and Throat (ENT) surgical services.

## Data sources and updated cost estimates

The data used for the cost-effectiveness analysis included estimates derived from personal communications with Deadly Ears staff, high-level summary data provided by Queensland Health or summarized from the Centre for Online Health database, data obtained from the published literature, and expert opinions. Tables 2 and 3 are updated here to include data source information and to clarify which values are based on assumptions. The revised version of Table 3 reports results of a reanalysis performed with updated information for Variable staff costs, Consumables, Variable travel and accommodation costs, Total annual cost, and Cost per surgery. The original analysis of costs, with data source information added, is included with this notice in the supporting information S1 Table. Fixed and variable costs in Table 3 and S1 Table are the annual average costs for outreach surgical services, and do not represent costs to the Cherbourg community or the Deadly Ears program.

Costings for the Deadly Ears Program ENT surgical services in S1 Table were based on estimates of resource use, staffing requirements, and the unit costs of those resources following discussion with staff at Cherbourg and running the MTESS and the Deadly Ears programs. These were not actual costings, but cost estimates, and were not provided by the Deadly Ears Program. Furthermore, since the analysis was conducted from a health system perspective, not all cost items included in S1 Table were incurred by the Deadly Ears program; they were actual costs incurred by other entities of the health care sector, including the State or Commonwealth Government.

An additional cost analysis was undertaken with early (2008) data previously supplied as expert opinions from the Deadly Ears staff (Table 3). The total cost for the MTESS was $236,200 vs. $78,243 for Deadly Ears (Table 2). Furthermore, the cost of employees who operate the programs were incurred to the health care system, but not specifically to the Deadly Ears or the MTESS programs. These include (i) the Aboriginal health worker employed full-time to conduct the mobile health screening program, and (ii) nurse and nurse unit manager employed to run the surgical service. The Deadly Ears Program did not employ any full-time staff in Cherbourg during the study period.

Travel costs included in the analysis were based on the assumptions that there would be 4 visits annually, each trip took 4 days, and assumed 8 working hours per day (4×4×8 = 128 hours per year). This is an error, per updated information the Deadly Ears Program made biannual visits. This is equivalent to Deadly Ears Program providing approximately 32 hours of ENT staff support in Cherbourg per year. The average cost per case was used in the analysis; if the number of visits and trips decrease, and there is a proportionate reduction in the number of cases, then the average cost per case would be similar to the estimate made by the authors. Fuel was estimated at $0.75 per km, in line with the Australian Tax Office claimable tax offset for the running costs of an average motor vehicle at that time.

## Updated sensitivity analyses

While costs in an economic evaluation are subject to variation, including due to temporal change and geographical variation, the impacts of variation in costing and related factors are addressed in sensitivity analyses. Reducing the staff costs for 25% (i.e., to the 32 annual hours, and 0.25 FTE for the Clinical Nurse and the Nurse Manager), plus adjusting the travel costs to reflect the two trips per year gives a total cost of $2,261 per surgery. The cost of surgery affects the total costs of both the Deadly Ears and the MTESS programs but remains relative. The additional sensitivity analysis (S2 Table) showed that at the cost of $2,261 per child surgery in the outreach clinic the MTESS remained cost-effective and provided better health outcomes (ICER = AU $507, compared to the original published ICER of AU $656 presented in Table 6 of [1]).

Additional sensitivity analyses were conducted after publication of [1] to further understand the impact of the cost of surgery on the conclusions pertaining to cost-effectiveness; this includes a table of results for the sensitivity analyses (S2 Table) and a new Tornado diagram (S1 Fig). The authors tested a claim that the actual cost was three to six times less than the cost presented in S2 Table (the surgical cost per case was $2,369). The authors replaced this number ($2,369 = $379,023 / 160 cases) with a cost value of $340 (which is more than six times smaller). They found that the (average) total costs (per strategy) for MTESS vs. Deadly Ears were $6,031 vs. $6,120 and QALYs were 15.644 vs. 15.902. Please note that these average total costs were not the one-off cost of providing surgeries or per screening occasion, but are the accumulated costs per individual (children) over a life time (of the model), which include screening cost, surgeries and treatment if it occurred, cochlear implants or other hearing aids and education cost for hearing loss children (if required).

Further probabilistic sensitivity analyses (PSA) were undertaken. In the original model, the authors specified the beta distributions using the base case value as mean, and variance as mean*0.2. For the revised model, the authors imposed new parameters to ensure all the beta distributions had a range as close to (0, 1) as practically possible. Since the authors specified the alpha and beta parameters from the base case value (as mean), they tested a range of variance values (for each beta distribution) to find the maximum variance values that still produce positive values for both alpha and beta parameters. The PSA results are presented on the willingness-to-pay spectrum in S2 Fig (original values) and S3 Fig (updated values).

With this Correction, the authors provide a revised version of Table 1 with additional information on the distributions used for the sensitivity analyses. In the probabilistic sensitivity analyses, the value for each parameter was drawn randomly from the respective distribution, then the model calculated the costs and outcomes for each strategy and the resulting incremental cost effectiveness ratio (ICER). The number of draws was 10,000 (same specification as the original analysis). The scatter plot of 10,000 draws is presented in S4 Fig.

## Discussion

There are a number of limitations made more explicit here.

The authors relied on expert opinion for some parameters. These parameters are subject to potential opinion bias, and the analysis of uncertainty may not fully reflect the variation that might be expected from expert opinion or assumptions used. The experts providing the information were experts in screening for hearing loss, ENT surgery, and indigenous health. In Tables 1 and 7, the authors specified the expert opinions and the sensitivity range based on their opinions. The range of some of the parameter values for the sensitivity analysis, such as “treatment failure” and “progression”, while not specified in Table 1, could be found in Tables 7 and 8 (and also available in the full model that is publicly available). Those parameters specified with a beta distribution, such as the “probabilities” are bounded within the range of 0 and 1. An updated sensitivity analysis is provided to address these concerns.

A point to highlight here is that there are more parameters derived from real-world data than assumptions used in the analysis. Like all economic evaluation models, it is informed by both actual data (trials) and literature; and this is the norm in economic evaluations. Program costs were calculated from the data provided within the studies, sourced from the literature, or where these sources did not contain the data required, expert opinions were sought. The scatterplot demonstrates the likely spread of the cost-effectiveness of MTESS compared with the service delivery model that was used prior to the introduction of MTESS (i.e., Deadly Ears Program, formerly called the Aboriginal and Torres Strait Islander Ear Health Program, during the years 2006–2008).

The limitation that this is a single site study is noted; evaluation of additional sites would be advantageous to confirm these results.

## Conclusions

Concerns were raised during the post-publication assessment that the conclusions reported in the article overstate what could be supported by the results, given the reliance of several parameters on expert opinion and/or assumptions, and in light of the PSA analysis results. This relates to the following:

- Tenth sentence of the Abstract: “We concluded that the MTESS service is a cost-effective strategy.”
- First and second sentences in the second paragraph of the Discussion section: “This model-based analysis shows that, compared to the Deadly Ears Program, the MTESS service is cost effective, with an average 98% probability of an acceptable ICER at the $50,000/QALY threshold. The cost effectiveness arises from preventing hearing loss in the given population and subsequent reductions in associated educational support costs and hearing aids and equipment costs.”
- First sentence of the Conclusion section: “…from a health service perspective, the supplemental mobile telemedicine-enabled screening and surveillance (MTESS) service is cost effective compared to the current practice alternative alone.”

The conclusions are hereby amended to state that the results of this study suggest that MTESS is likely to be a cost-effective strategy, but in light of the limitations discussed above and in [1] due caution should be exercised when making policy decisions.

## Tables

Please see the updated Tables 1–3 here.

**Table 1:** Transition probabilities.

	Base case	Sensitivity	Sources
Developing ear problems	Age dependent	Beta distribution, mean as base case, and standard derivation of 20% both sides.	ABS 2014 data
Being screened by Deadly Ears	0.39	Beta distribution, α = 0.300; β = 0.557	Queensland Ferret database
Being screened with MTESS service	0.80	Beta distribution α = 0.450; β = 0.113	Elliot et al 2009;
Screening returns false negative (diagnosed no ear problem given having ear problem)	0.05	Triangular distribution, range 0.02–0.20	Expert opinion
Screening returns true negative (diagnosed no ear problem given normal hearing)	0.90	Triangular distribution, range 0.80–0.95	Expert opinion
Getting treatment if diagnosed or have obvious sign of ear problem	0.86	Beta distribution α = 0.720; β = 0.119	Burns et al 2013; ABS 2014
Receive medical treatment (instead of surgical treatment)	0.80	Triangular distribution, range 0.70–0.90	Assumption
Treatment failure (both medical and surgical)	0.10	Beta distribution α = 0.044; β = 0.396	Expert opinion
Progression from ear problems to hearing loss without treatment	0.10	Beta distribution, α = 0.125; β = 1.125	Expert opinion
Getting hearing aids in Indigenous children	0.05	Triangular distribution, range 0.02–0.15	Expert opinion
Getting hearing aids in Indigenous adults	0.35	Triangular distribution, range 0.10–0.50	Expert opinion

**Note:** All the beta distributions are specified with a mean as base case, and a variance was based on plausible ranges for the parameters rather than including extreme values; plausible ranges were based on expert opinion. Since we specified the alpha and beta parameters from the base case value (as mean), we tested a range of variance values (for each beta distribution) to find the maximum variance values that still produce positive values for both alpha and beta parameters, but still ensure all the beta distributions have a range as close to [0, 1] as practically possible.

**Table 2:** Costs of screening, both strategies.

	Unit	Unit cost	Annual equivalent cost	Data sources
**Deadly Ears Program (Option A)**				
** Fixed costs**			** **	
** **Screening equipment	5 years	$5,852	$1,352	Expert opinion, Deadly Ears service, 2008
** **Carry cases	2 years	$504	$271	
** Variable costs**			** **	
** **Health worker (1.0 FTE)	1 FTE	$73,238	$73,238	
** **Consumables	1 year	$1,076	$1,076	
** **Mileage reimbursement (3,075km)	3,075km ^(a)^	$0.8	$2,306	
** Total cost**	887 children ^(b)^	**$88**	**$78,243**	
**MTESS (Option B)**				
** Fixed costs**			** **	
** **Van, fit-out and equipment	5 years	$192,298	$44,416	Expert opinion, Deadly Ears service, 2008
** **Garage	5 years	$23,256	$5,372	
** **Database costs	5 years	$50,236	$11,603	
** Variable staff costs**			** **	
** **Health worker	2 FTEs	$73,238	$146,746	Expert opinion for resources, Queensland Health Enterprise Bargaining Agreement 2012 for costs.
** **Senior ENT surgeon	169 hours ^(c)^	$121	$20,495	
** Variable travel, network and consumable costs**			** **	
** **Petrol (6,150km)	6,150 km ^(d)^	$0.8	$4,613	Standard costs based on the Australian Taxation Office, Internet Provider services and Queensland Health consumables.
** **Broadband wireless Internet access	12 months	$165	$1,980	
** **Clinical supplies	1 year	$1,246	$1,246	
** Total cost**	2026 children ^(e)^	**$117**	**$236,200**	

(a) Estimated travel distance required to cover 44% of the given population with an average weekly millage of 75km

(b)Assuming 35% of the estimated 2,533 registered children in the community are screened every 12-months

(c)Estimated total time to review 2026 screening assessments conducted in 1-year with an average review time per screen of five minutes

(d) Estimated travel distance required to cover 80% of the given population with an average weekly millage of 75km

(e) Assuming 80% of the estimated 2,533 registered children in the community are screened every 12-months

**Table 3:** Costs for surgical treatment in outpatient clinic.

	Unit	Unit cost	Annual equivalent cost	Data Sources
**Fixed costs**				
** **Anesthetic machine	5 years	$68,000	$15,706	Expert opinion, Deadly Ears service, 2008
** **Anesthetic monitor	5 years	$38,000	$8,777	
** **Additional anesthetic equipment (include nerve stimulator, portable suction unit)	2 years	$2,611	$1,404	
** **Patient monitor	3 years	$9,685	$3,556	
** **Miscellaneous equipment	3 years	$9,011	$3,309	
** **Surgical instruments	10 years	$74,331	$9,626	
** **Microscope	10 years	$14,497	$1,877	
** **Sterilizer	3 years	$6,540	$2,402	
** **Carry cases	2 years	$1,846	$993	
** **Clinic instruments	5 years	$2,086	$482	
**Variable staff costs (QH certified enterprise bargaining agreement) 2012**				
** **Nurse manager (per annum)	0.25 FTE	$98,153	$24,538	Expert opinion, Deadly Ears service, 2008. Costs for labour-based Queensland Health Enterprise Bargaining Agreement 2012 Hours of work updated to reflect the 32 hours (compared to 128 hours)
** **Clinical nurse	0.25 FTE	$79,992	$19,998	
** **Senior ENT surgeon	32 hours	$121	$3,881	
** **ENT registrar	32 hours	$83	$2,643	
** **Senior anesthetic consultant	32 hours	$118	$3,766	
** **Anesthetic registrar	32 hours	$80	$2,559	
** **Anesthetic technician	32 hours	$56	$1,799	
** **Scrub/scout nurses	32 hours	$50	$1,591	
** **Recovery room nurse	32 hours	$50	$1,591	
**Consumables**				
** **Anesthetic consumables and general supplies	2 trips	$1,180	$2,360	Expert opinion, Deadly Ears service, 2008
** **Anesthetic drugs	2 trips	$1,450	$2,900	
** **Surgical consumables	2 trips	$3,145	$6,290	
**Variable travel and accommodation costs**				
** **Truck rental (2 x 4 day trips)	8 days	$189	$1,512	Calculation was based on 2 trips per year (vs 4 trips) and cost based on expert opinion, Deadly Ears service, 2008.
** **Passenger van rental (2 x 4 day trips)	8 days	$137	$1,096	
** **Petrol (2 x 560km);	1120 kms	$1	$840	
** **Accommodation (2 x 4 night stays for 5 single rooms)	2 trips	$3,000	$30,000	
** **Meal allowance (2 x 4 day trips for 5 people at $70/day)	2 trips	$2,539	$25,390	
**Total annual cost**			**$180,888**	
**Cost per surgery (estimated 80 cases performed per year)**			**$2,261.11**	

## Supporting information

S1 Table Costs for surgical treatment in outpatient clinic. (DOCX)

S2 Table Additional sensitivity analyses for the cost per child. (DOCX)

S1 Fig One-way sensitivity analysis–Tornado diagram (ICER is in AU$ 2013–14). (TIFF)

S2 Fig Acceptability curve for different WTP threshold: Deadly Ears vs. MTESS (original values). (TIFF)

S3 Fig Additional PSA with wider variance specification for all beta distributions *(Note*: *as wide as permitted*, *that is to ensure the shape parameters are positive)*. (TIFF)

S4 Fig Additional probability sensitivity analysis results using second-order Monte Carlo simulation (10,000 samples), *using updated beta distribution specification*. (TIFF)
