# Supplementary material for: Correction: Cost-effectiveness analysis of a mobile ear screening and surveillance service versus an outreach screening, surveillance and surgical service for indigenous children in Australia
Source: PLoS One. 2020 Jun 5;15(6):e0234021. doi: 10.1371/journal.pone.0234021 (PMC7274379; doi:10.1371/journal.pone.0234021)
Supplement: S1 Table — (DOCX) [file pone.0234021.s001.docx]

**S1 Table. Costs for surgical treatment in outpatient clinic – *reproduced of Table 3, original publication.***

|  | **Unit** | **Unit cost** | **Annual equivalent cost** | **Data sources** |
| --- | --- | --- | --- | --- |
| **Fixed costs** |  |  |  |  |
| Anaesthetic machine | 5 years | $68,000 | $15,706 | Expert opinion, Deadly Ears service, 2008 |
| Anaesthetic monitor | 5 years | $38,000 | $8,777 |  |
| Additional anaesthetic equipment | 2 years | $2,611 | $1,404 |  |
| Patient monitor | 3 years | $9,685 | $3,556 |  |
| Miscellaneous equipment | 3 years | $9,011 | $3,309 |  |
| Surgical instruments | 10 years | $74,332 | $9,626 |  |
| Microscope | 10 years | $14,497 | $1,877 |  |
| Sterilizer | 3 years | $6,540 | $2,402 |  |
| Carry cases | 2 years | $1,847 | $993 |  |
| Clinic instruments | 5 years | $2,086 | $482 |  |
| **Variable staff costs (QH certified enterprise bargaining agreement 2012)** | | |  |  |
| Nurse manager (per annum) | 1 FTE | $98,153 | $98,153 | Expert opinion, Deadly Ears service, 2008. Costs for labour based on Queensland Health Enterprise Bargaining Agreement 2012 |
| Clinical nurse | 1 FTE | $79,992 | $79,992 |  |
| For labour Senior ENT surgeon | 128 hours | $121 | $15,523 |  |
| ENT registrar | 128 hours | $83 | $10,574 |  |
| Senior anaesthetic consultant | 128 hours | $118 | $15,066 |  |
| Anaesthetic registrar | 128 hours | $80 | $10,236 |  |
| Anaesthetic technician | 128 hours | $56 | $7,198 |  |
| Scrub/scout nurses | 128 hours | $50 | $6,365 |  |
| Recovery room nurse | 128 hours | $50 | $6,365 |  |
| **Consumables** |  |  |  |  |
| Anaesthetic consumables |  |  | $8,700 | Expert opinion, Deadly Ears service, 2008 |
| Anaesthetic drugs |  |  | $8,700 |  |
| Surgical consumables |  |  | $15,500 |  |
| **Variable travel and accommodation costs** |  |  |  |  |
| Truck rental (4 x 5 day trips) | 20 days | $189 | $3,780 | Deadly Ears service (for resource use). Costs applied based on the Australian Taxation Office and typical (estimated) costs for accommodation in rural areas. |
| Passenger van rental (4 x 5 day trips) | 20 days | $137 | $2,740 |  |
| Petrol (4 x 560km); | 2,240 km | $0.8 | $1,680 |  |
| Accommodation (4 x 4 night stays for 12 single rooms) | 192 nights | $110 | $21,120 |  |
| Meal allowance (4 x 5 day trips for 12 people at $70/day) | 240 days | $80 | $19,200 | Queensland Health Enterprise Bargaining Agreement 2012 |
| **Total annual cost** |  |  | **$379,023** |  |
| **Cost per surgery (estimated 160 cases performed per year)** |  |  | **$2,369** |  |
