# Supplementary material for: Correction: Cost-effectiveness analysis of a mobile ear screening and surveillance service versus an outreach screening, surveillance and surgical service for indigenous children in Australia
Source: PLoS One. 2020 Jun 5;15(6):e0234021. doi: 10.1371/journal.pone.0234021 (PMC7274379; doi:10.1371/journal.pone.0234021)
Supplement: S2 Table — (DOCX) [file pone.0234021.s002.docx]

**S2 Table. Additional sensitivity analyses for the cost per child.**

|  |  |  | **Strategy cost per child** | |  |
| --- | --- | --- | --- | --- | --- |
|  | **Total surgery cost** | **Surgery cost per child** | **Deadly Ears** | **MTESS** | **ICER** |
| Original estimate  (4 trips per year, 128 hours annually) | $379,023 | $2,369 | $6,235 | $6,262 | $656 |
| **Updated estimates** |  |  |  |  |  |
| 1. Staff hours reduced to 25% of the original estimates, updated other costs | $180,888 | $2,261 | $6,228 | $6,250 | $507 |
| 2. Surgery cost per child of $340  (6 times less than the original value) | | $340 | $6,120 | $6,031 | MTESS dominant |
| 3. Additional ranges of values of surgical cost per child |  |  |  |  |  |
| $500 |  | $500 | $6,129 | $6,049 | MTESS dominant |
| $1,000 |  | $1,000 | $6,157 | $6,106 | MTESS dominant |
| $1,500 |  | $1,500 | $1,686 | $6,163 | MTESS dominant |
| $2,000 |  | $2,000 | $6,220 | $6,214 | $148 |
| $2,500 |  | $2,500 | $6,277 | $6,242 | $836 |
| $3,000 |  | $3,000 | $6,334 | $6,270 | $1,524 |
