# Supplementary figures and images for: Correction: Cost-effectiveness analysis of a mobile ear screening and surveillance service versus an outreach screening, surveillance and surgical service for indigenous children in Australia
Source: PLoS One. 2020 Jun 5;15(6):e0234021. doi: 10.1371/journal.pone.0234021 (PMC7274379; doi:10.1371/journal.pone.0234021)

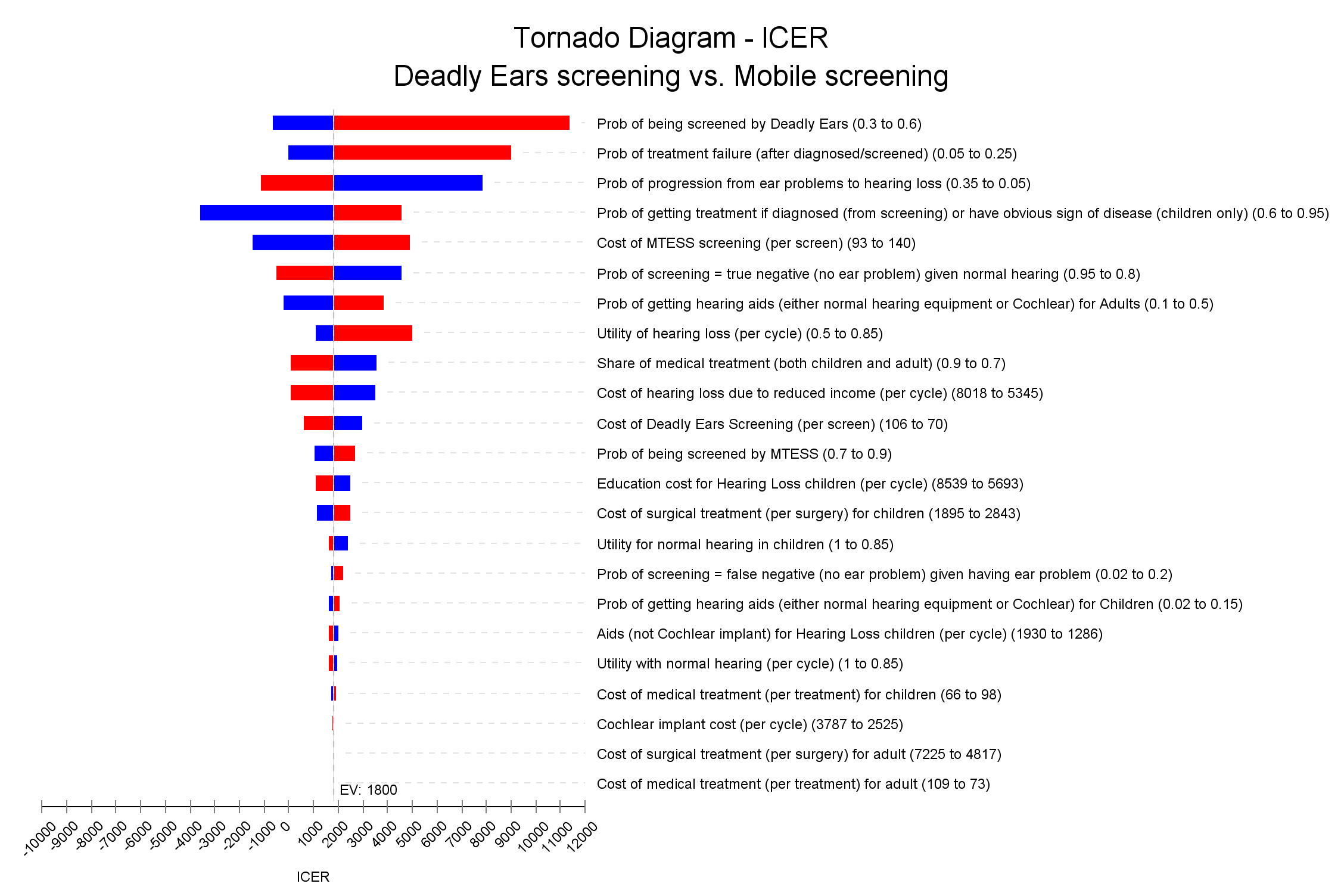

Supplement: S1 Fig — (TIFF) [file pone.0234021.s003.tiff]

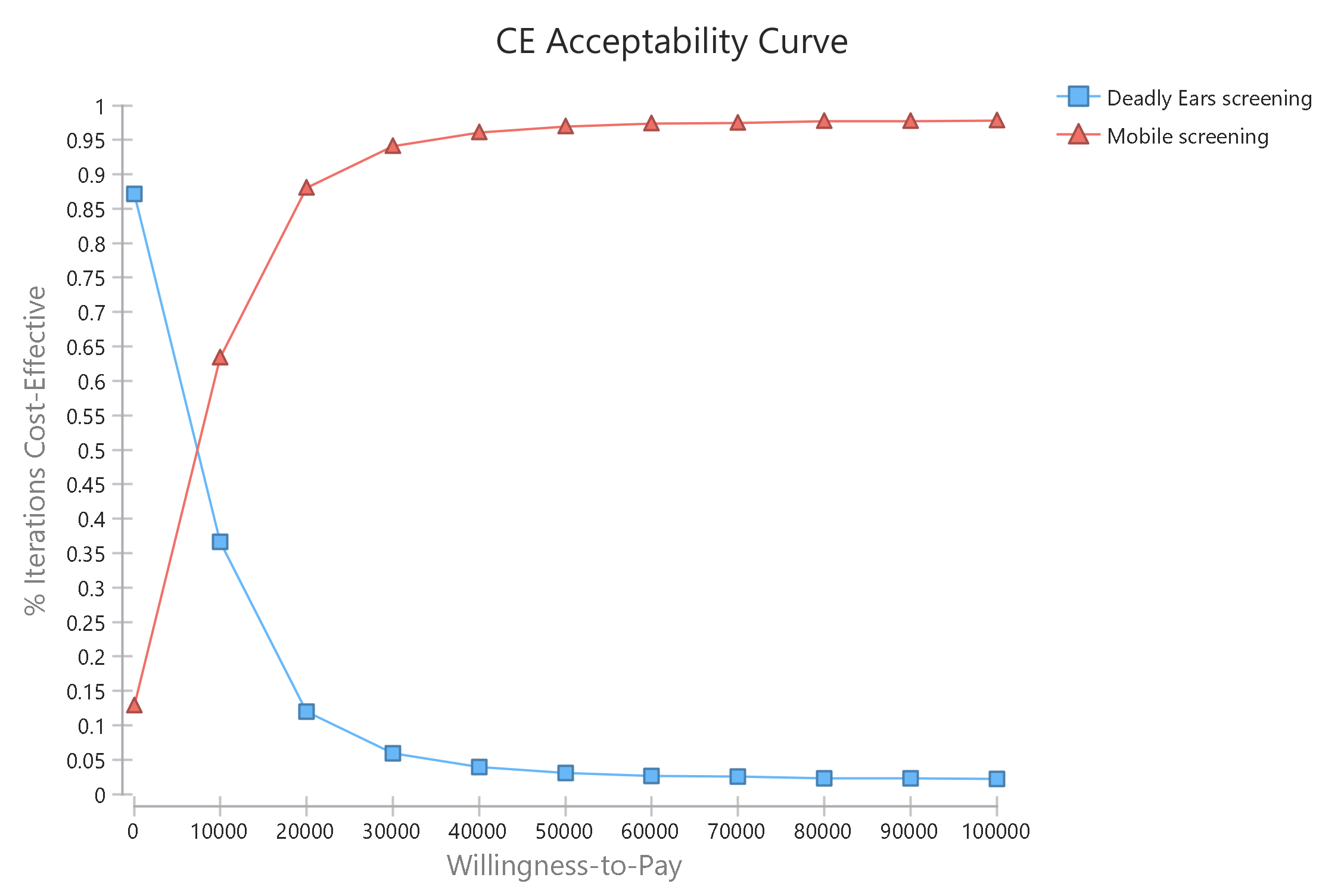

Supplement: S2 Fig — (TIFF) [file pone.0234021.s004.tiff]

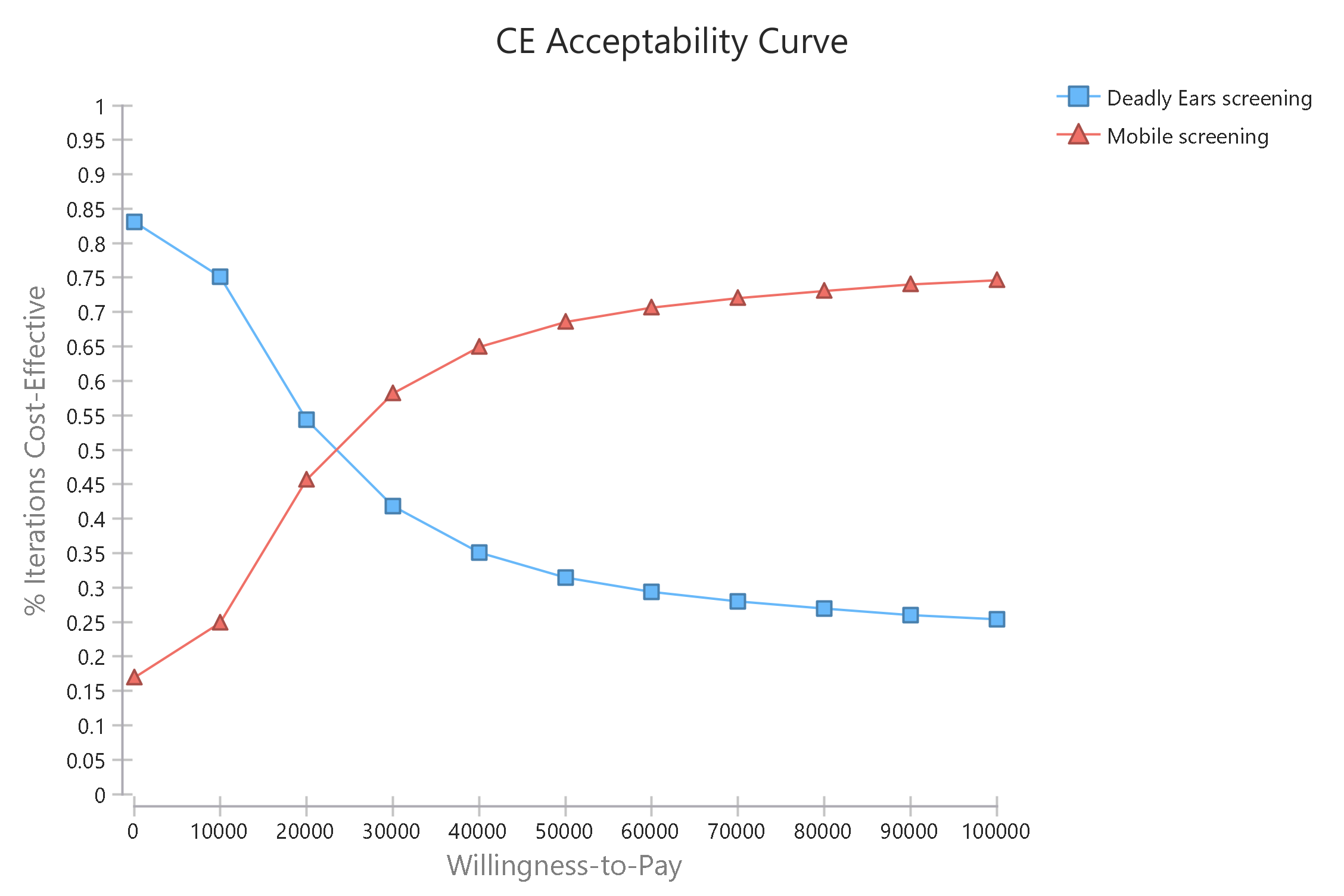

Supplement: S3 Fig — (TIFF) [file pone.0234021.s005.tiff]

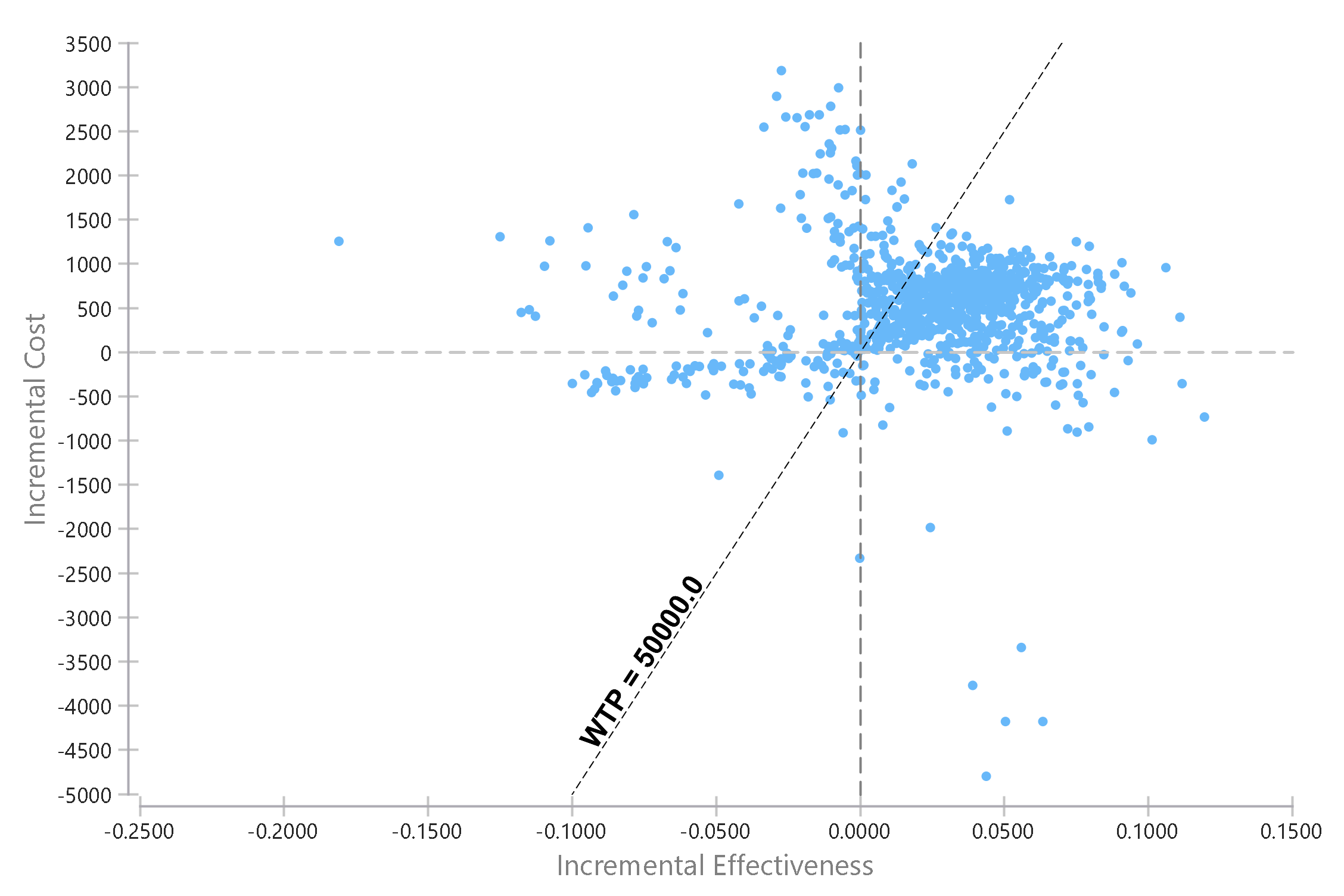

Supplement: S4 Fig — (TIFF) [file pone.0234021.s006.tiff]
